# The panorama of miRNA-mediated mechanisms in mammalian cells

**DOI:** 10.1007/s00018-013-1551-6

**Published:** 2014-01-29

**Authors:** Anna Stroynowska-Czerwinska, Agnieszka Fiszer, Wlodzimierz J. Krzyzosiak

**Affiliations:** Department of Molecular Biomedicine, Institute of Bioorganic Chemistry, Polish Academy of Sciences, ul. Noskowskiego 12/14, 61-704 Poznan, Poland

**Keywords:** miRNA, miRNA-mediated regulation of gene expression, Argonaute proteins, miRISC assembly, miRNA binding sites

## Abstract

MicroRNAs comprise a large family of short, non-coding RNAs that are present in most eukaryotic organisms and are typically involved in downregulating the expression of protein-coding genes. The detailed mechanisms of miRNA functioning in animals and plants have been under investigation for more than decade. In mammalian cells, miRNA guides the effector complex miRISC to bind with partially complementary sequences, usually within the 3′UTR of mRNAs, and inhibit protein synthesis with or without transcript degradation. In addition to these main mechanisms, several other modes of miRNA-mediated gene expression regulation have been described, but their scale and importance remain a matter of debate. In this review, we briefly summarize the pathway of miRNA precursor processing during miRNA biogenesis and continue with the description of the miRISC assembly process. Then, we present the miRNA-mediated mechanisms of gene expression regulation in detail, and we gather information concerning the proteins involved in these processes. In addition, we briefly refer to the current applications of miRNA mechanisms in therapeutic strategies. Finally, we highlight some of the remaining controversies surrounding the regulation of mammalian gene expression by miRNAs.

## Introduction

MicroRNAs (miRNAs) constitute a large family of short, non-coding RNAs (ncRNAs) (~22 nucleotides long) that are common in single-celled eukaryotes, plant and animal cells [1], and have also been found in virus genomes [2, 3]. In humans, more than 2,500 miRNAs have already been discovered (collected in the miRBase 20.0 database) [4].

MicroRNAs play a crucial role in the post-transcriptional regulation of gene expression, mostly involving gene silencing. These tiny RNAs are generated in cells through a biogenesis pathway that involves the two-step enzymatic processing of genome-encoded primary miRNA transcripts into short miRNA/miRNA* duplexes. During assembly of the miRNA-induced silencing complex (miRISC), the miRNA/miRNA* duplex is loaded into the Argonaute protein (Ago), and one of the strands (called the “passenger strand”) is released and degraded. Consequently, the bound miRNA strand (the “guide strand”) guides miRISC to interact with partially complementary sequences in target transcripts (mostly localized within the 3′UTR) and mainly triggers mRNA deadenylation and degradation or translation inhibition. However, several other mechanisms of gene expression regulation by miRNAs have also been described. The key attribute of miRNA is imperfect base pairing within miRNA/miRNA* duplexes as well as within duplexes formed by the miRNA and its target mRNA. It is estimated that most protein-coding genes are regulated by miRNAs [5, 6]. Thus, it is not surprising that miRNA deregulation influences cell physiology and triggers numerous pathological states.

In this review, after gathering the relevant information on miRNA biogenesis and miRISC assembly, we focus on the recent understanding of cellular mechanisms of gene expression regulation by miRNAs. This topic still arouses researchers’ curiosity because of the controversies around a detailed scheme of miRNA-mediated mechanisms.

## Overview of miRNA biogenesis

**Fig. 1:**
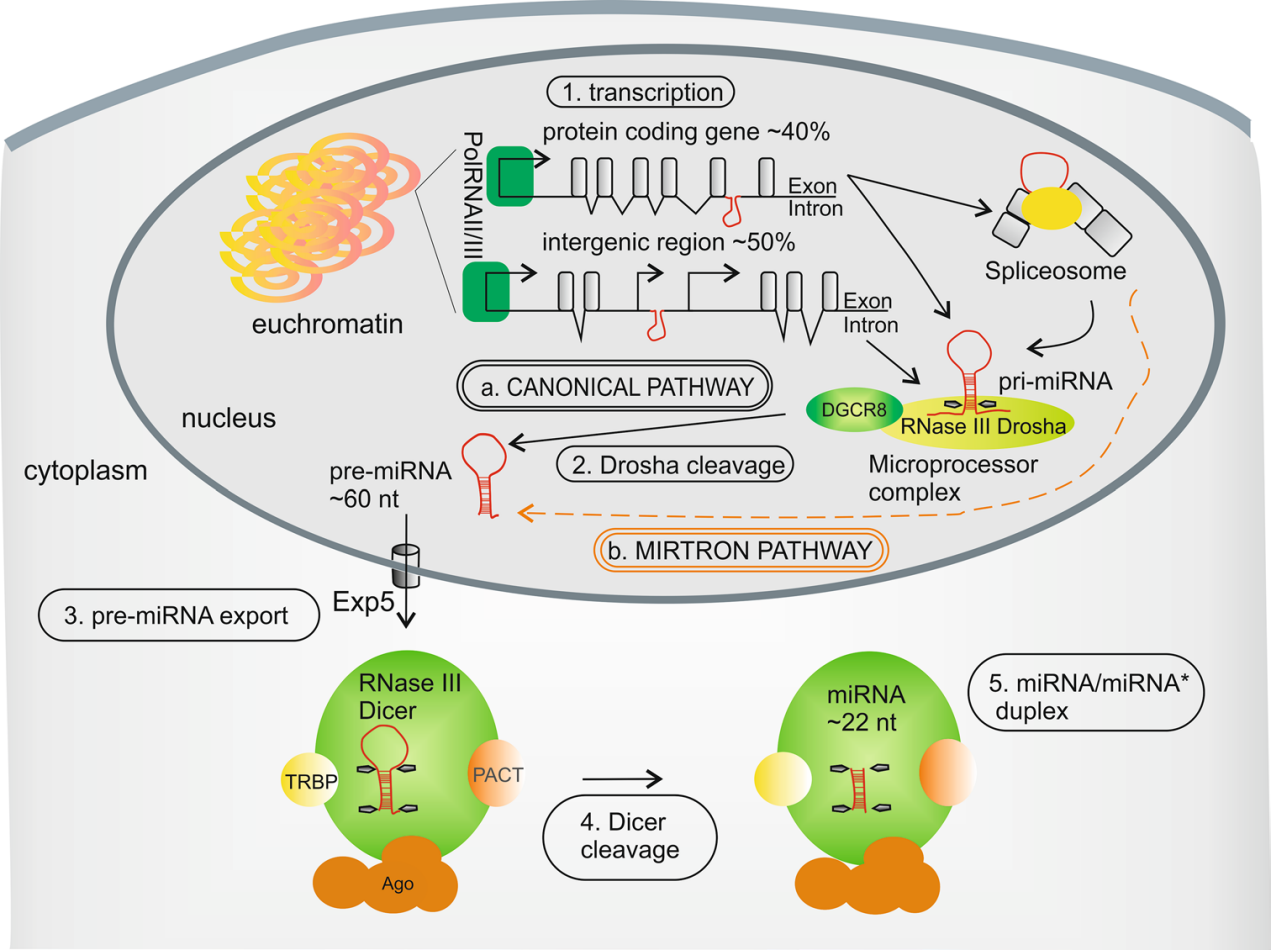
miRNA biogenesis in human cells. *a* The canonical pathway covers *1* pri-miRNA transcription, *2* Drosha cleavage, *3* pre-miRNA export to cytoplasm and *4* Dicer cleavage into *5* the miRNA/miRNA* duplex. *b* The alternative Drosha-independent biogenesis pathway (mirtron pathway) is indicated by the *orange dashed line*. See text for more details

The canonical biogenesis pathway of mammalian miRNAs (Fig. 1a) is a two-step (nuclear and cytoplasmic) enzymatic processing of their precursors, which are encoded in the genome (reviewed in [7, 8]). miRNA genes are localized either between protein-coding genes or in their intron elements. Briefly, miRNA genes are typically transcribed by RNA polymerase II [9, 10], or less frequently by RNA polymerase III (Fig. 1.1) [11], to produce long primary miRNA transcripts (pri-miRNA) containing a stem-loop structure. Pri-miRNAs are co-transcriptionally recognized by a large protein complex, called the Microprocessor, the main components of which are the RNase III Drosha and DGCR8 [12–16]. Drosha is responsible for pri-miRNA cleavage—in a process called “cropping”—into ~60 nucleotides long, hairpin-structured pre-miRNA (Fig. 1.2) [12, 17]. Next, pre-miRNAs are actively transported from the nucleus into the cytoplasm by Exportin-5 (Exp-5) (Fig. 1.3) [18, 19]. In the cytoplasm, another RNase III, Dicer, functioning with its protein partners Ago2, TRBP and/or PACT [20–24], recognizes the pre-miRNAs and cleaves them into miRNA/miRNA* duplexes of approximately 22-nucleotide length in a process termed “dicing” [25–28] (Fig. 1.4, 5). In addition to the canonical pathway of miRNA biogenesis described above (Fig. 1a), other pathways exist that are independent of Drosha (wherein miRNAs are generated from pre-miRNA-like introns, called mirtrons) (Fig. 1b) [29, 30] or Dicer activity [31–33].

RNase III enzymes (Drosha and Dicer) cleave miRNA precursors imprecisely and generate heterogeneous products that often differ in length [28, 34–36]. These length variants and shifted miRNA sequences are called isomiRNAs (isomiRs) [34]. The isomiRs may vary in their regulation of gene expression because of changes in their target specificity (reviewed in [37]). Some isomiRs, however, may regulate the same targets as the canonical miRNAs [38, 39].

Apart from isomiRs, the pool of miRNAs may be varied by the competitive processes of post-transcriptional, non-templated oligo-adenylation [35, 40–42] or oligo-uridylation [43] at the 3′ termini of pre-miRNAs ([44], reviewed in [45, 46]). In general, the addition of the U-residues to the 3′ end of RNAs serves as a molecular signal for degradation ([43, 47–49], reviewed in [50]), whereas the extra A-residues tend to increase miRNA stability [41]. Moreover, miRNA precursors might be substrates for adenosine deaminase acting on RNA (ADAR), which converts A-residues into inosine, causing A-to-I RNA editing [51–53]. Inosine pairs with C- instead of U-residues; hence, it may alter the structure and properties of modified RNA. As a consequence of miRNA precursor sequence modifications by ADAR, several effects may occur, such as miRNA processing inhibition [52, 54, 55], miRISC loading repression [56] or changes in the repertoire of the targeted genes (redirection of targets) ([57], reviewed in [58]).

## miRISC assembly

**Fig. 2:**
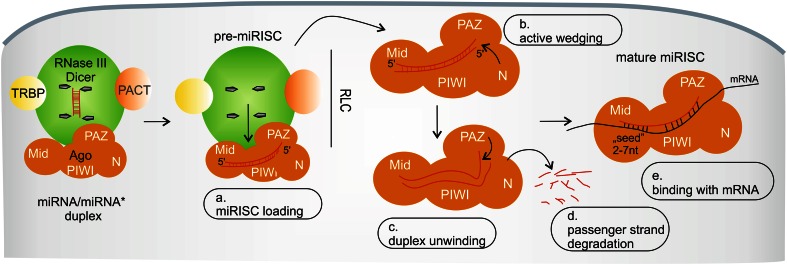
miRISC assembly in human cells. *a* The first step is miRISC loading, when the miRNA/miRNA* duplex is transferred from Dicer to Ago in the miRISC loading complex (RLC). *b* Next, domain N of Ago actively wedges between miRNA strands and *c* the PAZ domain of Ago unwinds the miRNA duplex. *d* The passenger strand is removed from miRISC and undergoes rapid degradation. *e* miRNA within mature miRISC binds with imperfect complementarity to its target sites. See text for more details

miRNA/miRNA* duplexes are incorporated into a ribonucleoprotein (RNP) complex—called miRISC—that plays a crucial role in the miRNA-mediated mechanism of gene expression regulation. The core component of miRISC is a protein from the Argonaute subfamily, which is characterized in human cells by the existence of four paralogs: Ago1-4 (reviewed in [59, 60]). Ago proteins consist of four domains: PAZ (binds the 3′ end of the miRNA strand), Mid (binds the 5′-phosphate group of the miRNA), C-terminal PIWI (may possess endonucleolytic activity) and the N-terminal domain (facilitates duplex unwinding) (Fig. 2a). Earlier findings regarding the roles of Ago domains [61–65] have been recently confirmed by the determination of the three-dimensional structure of human Ago2 alone [66] and in a complex with miR-20a [67]. Among the Ago paralogs, only Ago2 (also called “Slicer”) is characterized by siRNA-induced endonuclease activity toward complementary mRNA sequences [61, 68–70] because of the specific structural [69, 71] and functional [72, 73] features of its PIWI domain, which is similar to that observed in RNase H [74]. As it was discovered that miRNAs and siRNAs in *Drosophila melanogaster* cells are sorted into different Ago paralogs ([75–78], reviewed in [79]), it was thought that such sorting may also occur in mammalian cells. However, no miRNA preference for a particular Ago protein paralog has been found by the deep sequencing of miRNAs bound to immunoprecipitated Ago proteins [80]. In addition, according to shotgun proteomic analysis, miRNAs are sorted randomly depending on the abundance of Ago paralogs [81].

**Table 1:** The proteins involved in miRNA-mediated mechanisms of gene expression regulation and their interactors

Protein	Role	Interactors (interacting protein domain)	miRNA-mediated mechanism of gene expression regulation	References
Ago1–4 (Argonaute)	Core component of miRISC, miRNA binding	TNRC6 (PABC domain), FMRP, Imp8, HuR	Translation inhibition or upregulation, transcript deadenylation and decapping	[59, 82, 85]
TNRC6A–C (GW182, GW-repeat containing protein)	Core component of miRISC and P/GW-bodies; linker between PABP and deadenylases	Ago (GW/WG-repeats), PABP (PAM2 domain), EDD (PAM2 domain), PAN3 (CIM2 region),	Translation inhibition or upregulation, transcript deadenylation and decapping	[196, 201, 262, 263]
TNR6A	Ago2-navigator protein	CNOT1 (CIM1/CIM2 region), Exp1 (NES)	Gene silencing in the nucleus	[245]
PABPA–C (polyA-binding protein)	Binding to polyA tail of transcript; interaction with initiation factors	TNRC6 (PABC domain), PAN3 (PABC domain)	Translation inhibition, transcript deadenylation	[264]
PAN2–PAN3 complex:			Initial transcript deadenylation	
PAN2 (polyA nuclease)	Nuclease activity	PAN3		[228]
PAN3	Binding to other proteins	PABP (PAM2 domain), TNRC6 (PAM2 domain), PAN2		[201, 232]
Multisubunit CCR4-NOT complex:			Transcript deadenylation	
CNOT1	Scaffold for CCR4-NOT complex	TNRC6, Caf1, CNOT4, CNOT2/3, CNOT 9/10		[200–202]
Caf1a/b (CNOT7/8)	Deadenylase subunit	CNOT1		[233]
Ccr4a/b (CNOT6/6L)	Deadenylase subunit	CNOT1, Caf1 (leucin-rich region, LRR)		[228, 265, 266]
Tob	Association of CCR4-NOT	Caf1, PABP (PAM2 domain), EDD	Transcript deadenylation	[264]
DCP1-DCP2 complex:			Transcript decapping	
DCP1 (decapping protein)	DCP2 activator	DCP2		[239, 267]
DCP2	Decapping subunit	DCP1, RCK/p54, LSm4, PNRC2		[239, 267, 268]
RCK/p54 or DDX6 (DEAD-box helicase)	DCP2 activator; responsibility for the P-body localization of DCP1-DCP2	DCP2, EDC3, Pat1, EDD	Cap-dependent translation repression, transcript decapping	[239, 269, 270]
EDC4 EDC3	Association between DCP1 and DCP2; stimulation of decapping	DCP2, RCK/p54	Transcript decapping	[270]
XRN1	5′-3′ exoribonuclease activity	Pat1	Transcript degradation	[271]
FMRP	Stress granule component	Ago1, Ago2	Translation regulation	[221, 244, 272, 273]
LSm4	P-body component, decapping activator	DCP2	Transcript degradation	[244]
PNRC2	Synergy with DCP1, decapping stimulation	DCP2	Transcript decapping	[267, 274]
Pat1	DCP2 activator, linker between deadenylation and decapping, induce the formation of P-bodies	DCP2, XRN1, RCK/p54	Transcript decapping	[271]
EDD	E3 ubiquitin ligase	TNRC6 (PABC domain), RCK/p54, Tob	Translation inhibition and transcript deadenylation	[275]
Imp8 (Importin-8)	Transport to the nucleus	Ago	Translation inhibition	[244]
Exp1 (Exportin-1)	Transport of TNRC6A from nucleus	TNRC6	Gene silencing in the nucleus	[245]
HuR	Binding to AU-rich elements (ARE-binding site) by three RRM (RNA recognition motifs)	HuR oligomerization	HuR-mediated derepression of miRNA-mediated gene expression; HuR-stimulated miRNA-mediated gene silencing	[223, 276, 277]

Mature miRISC is formed in a multistep assembly process (Fig. 2) (reviewed in [82]). The first step is miRISC loading and the formation of pre-miRISC, when the miRNA duplex is transferred from Dicer to Ago within the RISC loading complex (RLC); this process requires ATP [83–85] and the 5′ phosphate of miRNAs [83]. Interestingly, it has also been shown that miRISC loading might be preceded by miRNA precursor deposit complex (miPDC) formation [86]. The major components of RLC are Dicer, Ago, TRBP and/or PACT (Fig. 2a) [20, 21, 87, 88], and these proteins were shown to participate in strand selection [89–91]. However, the detailed composition of RLC is still under investigation, and it has been suggested that Dicer is not essential in asymmetric miRISC loading [92–94].

It has been shown that either strand from a duplex can be a guide or a passenger strand [95–99]. The selection of a guide strand is thought to depend on the relative thermodynamic stability of miRNA ends, referred to as the “asymmetry rule.” The strand with the less stable 5′ end base pairing in the duplex is typically retained by miRISC [100–102]. Moreover, on the 5′ end of the miRNA guide strands, A- and U-residues are much more preferable for the miRISC loading step than are G- or C-residues [103]. Other factors contributing to the efficiency of duplex loading and unwinding are structural features of the miRNA/miRNA* duplexes (e.g., the position of base mismatches) and their sequence composition [84, 85, 104]. Taken together, the differences and changes in the miRNA sequence, such as those observed in isomiRs or edited miRNAs, may affect guide-strand selection [105].

miRISC loading is thought to be continued with an active wedging of the Ago N-terminal domain between duplex strands (Fig. 2b) [106] and duplex unwinding by the Ago PAZ domain (Fig. 2c) [107]. As a result, one strand from a duplex is removed from pre-miRISC and undergoes rapid exonuclease-mediated degradation (Fig. 2d) (reviewed in [46]). Hence, the interaction of Ago proteins with miRNAs [67, 108–110] increases miRNA stability in the cell. Similarly, Ago cellular stability is enhanced by miRNA binding [67, 111].

### Alternative miRISC assembly process

It is commonly held that the Ago:miRNA ratio is approximately 1:1 and that the model of miRNA-mediated repression is stoichiometric rather than catalytic. However, Gagnon, Novina and colleagues recently proposed an alternative miRISC assembly [112]. Based on several lines of evidence (e.g., quantitative proteomics and RT-qPCR), the authors demonstrated an at least several-fold cellular excess of miRNA molecules relative to Ago1-4 proteins, and they proved the existence of Ago-free miRNAs [112]. Consistent with this result, other researchers have observed an excess of total miRNAs in comparison to Ago-bound miRNAs [113, 114]. Thus, it might be a general rule that Ago is a limiting factor and that miRNA needs to compete for loading into miRISC.

The proposed alternative RISC assembly pathway implies a catalytic mode of miRNA function, wherein Agos first anneal miRNA guide strands to target transcripts, dissociate from the miRNA-mRNA duplexes, bind another miRNA/miRNA* or miRNA-mRNA duplex, and finally trigger silencing activity (which is, however, less efficient for pre-annealed miRNA-mRNA than for the canonical pathway). This results in miRNA protection and cellular stabilization [115, 116] and allows for the association of multiple miRNAs to mRNAs. This pathway may explain the specificity of miRNA binding to sponges and competing endogenous RNAs (ceRNAs) (see Sect. “Modulation of miRNA interactome”) [112].

Importantly, the authors demonstrated direct interactions between Ago proteins and pre-annealed miRNA-mRNA duplexes [112]. Their result is not entirely unexpected, as several earlier in vitro studies showed Ago binding to pre-annealed miRNA-mRNA duplexes [117–119]. The flexible structure of Ago2 [67] may allow its loading with such a complex by appropriate spatial orientation of Ago domains. Together, the studies by Gagnon, Novina and colleagues shed new light on miRNA-mediated mechanisms, but the scale and importance of this alternative mechanism need to be determined.

## miRNA-binding sites and their identification

### Characteristics of miRNA-binding sites

In the canonical pathway, miRISC (guided by the single miRNA strand) finds partially complementary sequences in an mRNA and binds to the transcript (Fig. 2e). However, the detailed mechanism by which miRISC finds the miRNA target site is still unknown. It is thought to be a result of a diffusion-controlled process rather than transcript scanning by miRISCs [120, 121]. The efficiency of miRISCs binding to their targets was shown to depend on RNA structural factors, such as target sequence accessibility [122–126].

The occurrence of the minimal perfect Watson–Crick pairing between the miRNA and the targeted mRNA sequence is essential for miRNA-mediated gene expression regulation, at least in the specific region called the “seed” region (2–7 nucleotides at the 5′ end of the miRNA) (Fig. 2e) [127, 128]. Apart from the canonical pairing site, the rest of the sequence (the 3′ end) may form an additional matching region called the 3′-supplementary or 3′-compensatory site [129, 130], but nucleotides 9–12 tend to be mismatched to prevent Ago2-mediated cleavage of mRNAs [129]. The importance of “seed” pairing is well proven for the regulatory functions of miRNAs [67, 129]; thus, isomiRs with shifted 5′ ends may regulate different targets because of the changed “seed” sequence [35, 39, 96]. However, recent global analyses showed that only <40 % miRNA-target interactions involve uninterrupted Watson-Crick base pairing in the “seed” region [131]. Moreover, several non-canonical interactions within the “seed” region were found to be functional [131–134], which suggests that miRISC may be more flexible in target binding than previously anticipated.

**Fig. 3:**
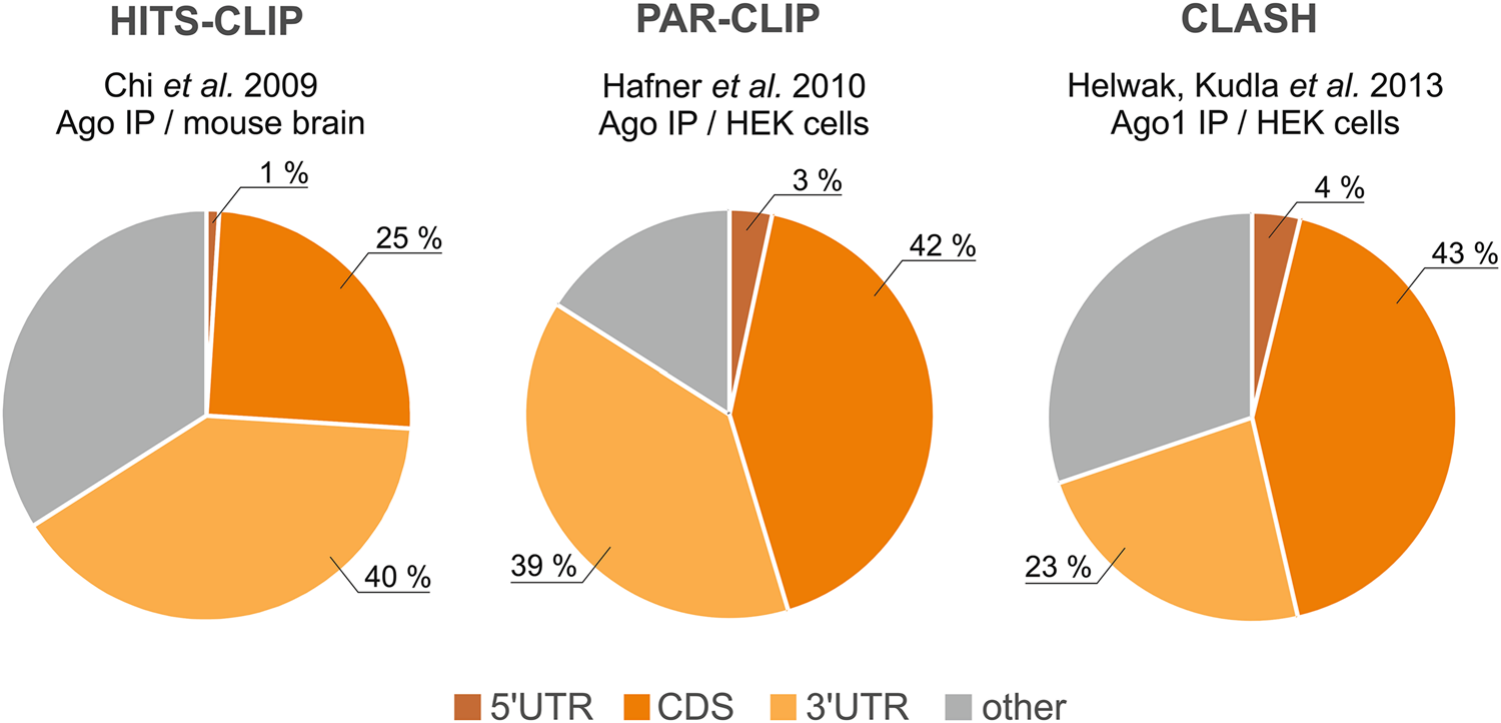
The miRNA-binding site distribution in the mammalian transcriptome as revealed by different global analyses of RNAs immunoprecipitated with Ago proteins. The method, reference and experimental basics are given for each analysis. The main miRNA targets are found in mRNAs and were mapped to 5′UTR, CDS and 3′UTR regions (approximate shares of these reads are given in the pie charts). A group of “other” reads contains different non-coding RNAs: pseudogenes, intronic and intragenic sequences

Until recently, functional miRNA-binding sites were thought to be usually localized within the 3′UTR [129] but might also be present within the coding sequence (CDS) [135–139] and the 5′UTR [140–142]. Surprisingly, the results from genome-wide analyses of miRNA-binding sites, performed by the Darnell, Tuschl, Sharp and Tollervey groups, clearly indicate that a substantial fraction of miRNAs interacts with sequences localized within the CDS (Fig. 3) [131, 143–145]. This suggests that the CDS may be widely targeted by miRNAs and that these miRNA target sequences might be underestimated. However, the functionality of the miRNA-binding sites in the 5′UTR and CDS is considered to be lower than that of the 3′UTR as miRISC may not resist the collision with the scanning small ribosomal subunit and rapidly translocating ribosomes [146].

Importantly, the efficiency of miRNA-mediated gene expression regulation may depend on the number of miRNA-binding sites within regulated targets [147, 148] and the distance between these sites [147, 149–151]. The more target sites that are present on the transcript, the higher the observed silencing efficiency. This phenomenon is likely a result of cooperative interactions between neighboring bound miRISCs [128, 147, 149, 151].

Taking the rules for miRNA binding to transcripts together, the most efficient target regulation involves multiple “seed”-containing interactions within the 3′UTR of an mRNA. Nevertheless, many non-canonical interactions were identified, and their functionality remains to be established.

### Prediction and identification of miRNA-binding sites

The reliable identification of transcripts that are regulated by miRNAs is essential for unraveling the specific cellular functions of miRNAs (reviewed in [152, 153]). Thus, several algorithms have been developed for miRNA target predictions including miRANDA [154], RNAhybrid [155, 156], PicTar [157], TargetScan [158] and PITA [159] (compared in [130, 160]). These algorithms take into account i.a. the relevance of “seed” pairing, the sequence conservation among species, the free energy of miRNA-mRNA binding and target site accessibility. In addition, new algorithms, for example, miRco (predicting binding sites in a cooperativity-permitting distance) [151] and MREdictor (accounting for protein-binding sites within miRNA target sequence) [161], have been described.

The predicted miRNA-mRNA interactions may contain many false positives; thus, the functionality of such interactions needs to be verified experimentally. The methods for validating miRNA targets include the use of miRNA inhibitors or miRNA mimics in dedicated gene-specific expression assays, usually luciferase reporter assays, or in high-throughput assays (reviewed in [162, 163]). Global methods developed in recent years enabled the analysis of a pool of Ago-bound miRNAs [80, 164] and Ago-bound transcripts [143–145] in mammalian cells. The basis for these analyses is the use of UV crosslinking and immunoprecipitation (CLIP) followed by deep sequencing and bioinformatic mapping of the reads ([165, 166], reviewed in [167]). Nevertheless, in global analyses of the RNA interactome, it is challenging to extract a single specific miRNA-mRNA interaction from the whole data set. More recently, a novel method of crosslinking, ligation, and sequencing of hybrids (CLASH) was developed [168] and applied for the high-throughput identification of miRNA-target pairs [131]. CLASH allowed the identification of ~18,000 high-confidence miRNA-target interactions (via Ago1) in human cells and provided more precise insights into the miRNA interactome than were possible previously (Fig. 3).

### Modulation of the miRNA interactome

Target regulation by miRNAs can be additionally controlled by modulation of miRNA-binding sites. Specific factors may increase or decrease the number of miRNA target sites and block or facilitate miRNA binding. For instance, the presence of several alternative polyadenylation sites (PAS) may give rise to transcripts that differ in the length of their 3′ sequence (reviewed in [169, 170]) and thus the number of miRNA-binding sites. The selection of the functional PAS may depend on the developmental stage of the cells or the tissue specificity. For example, miR-206 supresses the expression of Pax3 in limb muscle stem cells but not in diaphragm muscle stem cells, because in the latter cells 3′UTR of the Pax3 transcript is shortened that results in loosing miR-206-binding sites [171]. Furthermore, specific RNA-binding proteins (RBPs) (e.g., Dnd1 or Pumilio) may possess binding sites overlapping with miRNA binding sites, causing them to interfere with one another [172, 173]. Moreover, post-transcriptional modification of the transcript, i.a., deamination of A residues, may create novel miRNA-binding sites [174] or eliminate miRNA:mRNA recognition [175]. In addition, transcripts that share microRNA response elements (MREs) may co-regulate each other through the competitive binding of miRNAs [176, 177]. To address this issue, a hypothesis regarding competing endogenous RNAs (ceRNAs) was proposed. It suggests that both coding and noncoding RNA may crosstalk through MREs with miRNAs [178] and suppress the regulatory functions of miRNAs by sequestering them and affecting the pool of miRNAs available for target binding [179–181]. Thus far, different types of long non-coding RNAs (lincRNAs) [182] have been described as miRNA antagonists, including circular RNAs (circRNAs) [183, 184], pseudogenes [185] and viral RNAs [186]. Moreover, many other types of RNAs (e.g., rRNA and tRNA) were found to interact with miRNAs [131, 143], but the relevance of these binding sites remains to be determined.

## Variations in miRNA-mediated mechanisms

**Fig. 4:**
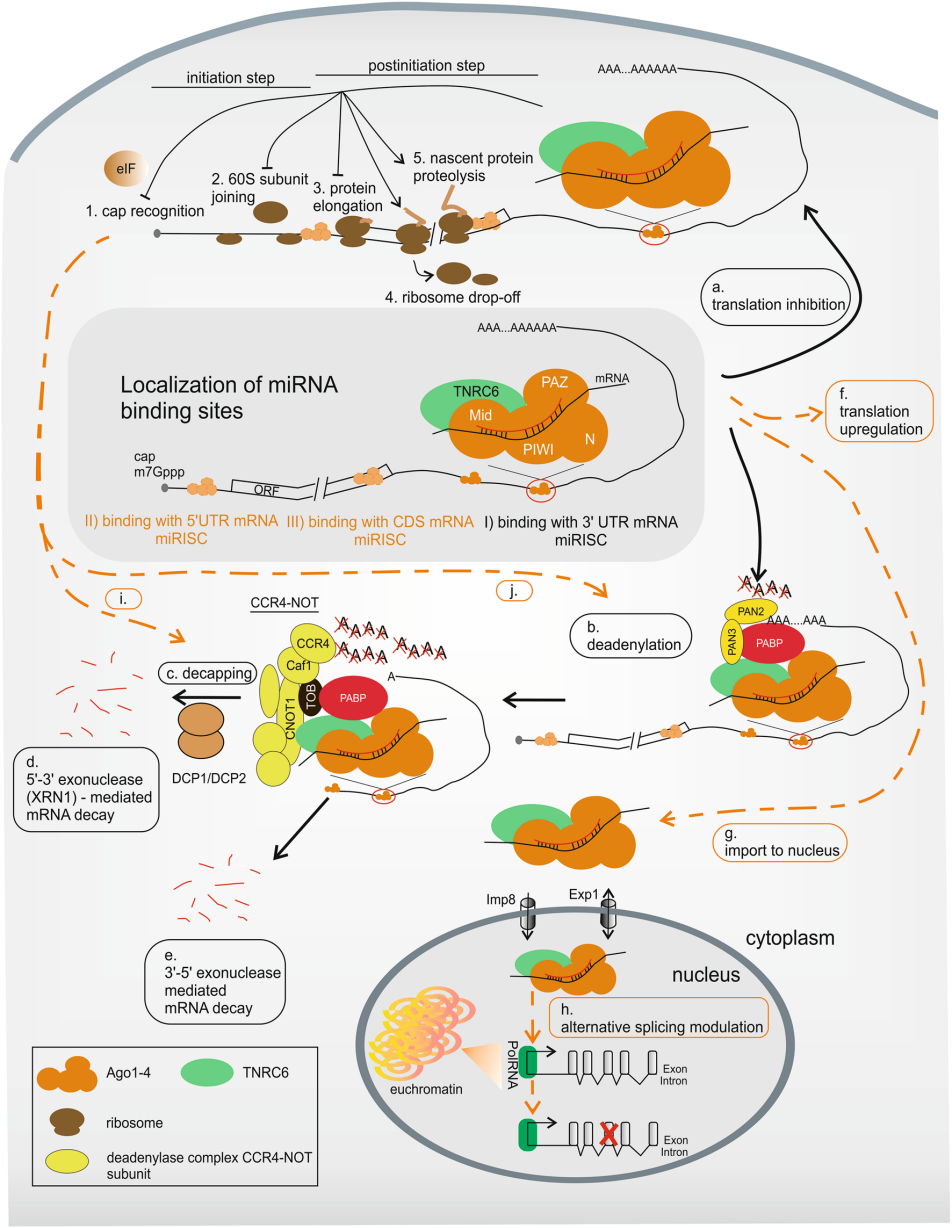
miRNA-mediated mechanism of gene expression regulation in human cells. Mature miRISC binds miRNA target sites localized *I* mostly within the 3′UTR but also (indicated in *orange*) *II* in the 5′UTR or *III* in the CDS. There are two main pathways of miRNA-mediated mechanisms of gene expression regulation: *a* translation inhibition either on initiation (*1*, *2*) or at a post-initiation step (*3*–*5*) and *b* deadenylation followed by *c* decapping and *d*, *e* mRNA decay. However, some less well-known alternatives, indicated by *dashed orange lines*, have been described: *f* translation upregulation, *g* import into the nucleus and *h* alternative splicing modulation, *i* decapping followed by translation inhibition and *j* deadenylation followed by translation inhibition. See text for more details

The miRISC binds to a targeted mRNA sequence and typically downregulates its translation mainly via mRNA deadenylation (Fig. 4b), which causes transcript decay (Fig. 4d, e) [187–189], or via translation inhibition (Fig. 4a) [190–192]. However, the detailed composition of miRISC, as well as its regulatory function, is still under investigation. The main proteins currently known to be involved in miRNA-mediated mechanisms are listed in Table 1.

It is postulated that at least two miRISC components, interacting with each other, are crucial in miRNA-mediated mechanisms: Argonaute (see the Sect. “miRISC assembly”) and conserved GW182 protein family members. GW182 is the main component of processing bodies (P-bodies or GW-bodies; see the Sect. “Transcript deadenylation and degradation”). Three paralogs of the GW182 protein (TNRC6A–C) are expressed in vertebrate cells (reviewed in [193]). Characteristic features of the GW182 protein family include many glycine-tryptophan or tryptophan-glycine repeats (GW/WG repeats) [194], the number and localization of which vary among family members. The N-terminal GW-repeats comprise multiple independent Ago-binding sites, called Ago hooks ([195–198], reviewed in [199]), whereas C-terminal GW repeats are the binding platform for the subunits of deadenylase complexes: NOT1 [200–202] and PAN3 [200]. Structurally, the vertebrate and insect proteins are composed of three main parts: the N-terminal Ago-binding domain, the central ubiquitin-associated (UBA)-like domain and the C-terminal silencing domain with the non-canonical RNA recognition motif (RRM), which is most likely responsible for the protein-protein interactions [203]. The silencing domain is believed to trigger gene silencing by both translational repression and mRNA decay [204–207]; it contains a PAM2 motif that interacts with the PABC domain of the polyA-binding protein (PABP). This motif allows GW182 to function as a link between Ago and other proteins (Table 1).

### Translation regulation: inhibition and rare upregulation

The first postulated miRNA-mediated mechanism was translation inhibition without transcript downregulation [190–192]. Protein synthesis inhibition is likely cap (m7G)-dependent, because the lack of a natural cap [208] or the presence of its analogs impairs this process [209]. However, some researchers have reported a mechanism of cap-independent, IRES-driven translation repression [210, 211]. Additionally, the translational step that is inhibited by miRNAs remains a matter of debate. The results of ribosome profiling experiments suggest the formation of both light, monosome- [208, 212] and heavy, active polysome-containing fractions [213, 214] on mRNA during miRNA-mediated gene silencing. These findings indicate that protein synthesis regulation may occur at either translation initiation (Fig. 4a, 1–2) or elongation (Fig. 4a, 3–5).

The blockage of translation initiation may be caused by defects in ribosome recruitment to the regulated mRNA. One explanation for such a blockage is the impairment of cap recognition resulting from the interference of miRISC-associated proteins with translation initiation factors (Fig. 4a, 1) [209, 215] or from the interaction of miRISC with the 5′ cap structure [212, 216]. The second possibility is the repression of 60S ribosome subunit joining (Fig. 4a, 2) [217, 218]. In contrast, the formation of polysomal fractions during miRNA-mediated silencing suggests an opportunity to inhibit protein synthesis at the post-initiation level [210, 213, 214]. This most likely involves a block in elongation (Fig. 4a, 3) [213], ribosome drop-off (Fig. 4a, 4) [210] or nascent protein proteolysis (Fig. 4a, 5) [191, 214]. However, the significance of these latter mechanisms seems to be rather limited.

According to the existing knowledge, translation initiation is extensively regulated (i.a., during mRNA circularization, cap recognition, initiation complex formation, 40S scanning); thus, intuitively, it may be easier to affect any of these regulatory points. Therefore, we favor miRNA-mediated translation inhibition at the initiation step rather than during elongation. Surprisingly, in the output of miRNA interactome revealed by CLASH, the most numerous and highly reproducible non-mRNA chimeras were formed with the 18S and 28S rRNAs, suggesting miRNA interactions with ribosomes [131]. This is consistent with previous evidence for the association of miR-206 with nuclear pre-ribosomes and cytoplasmic ribosomes [219] and for the presence of ribosomal proteins in the Ago-associated protein fraction [220]. Such an interaction may imply that miRISCs directly interfere with ribosomes and trigger translation inhibition.

Interestingly, under certain cellular conditions, such as cell cycle arrest in G0/G1, cellular stress or nutrient shortage, miRNAs may also induce translation upregulation (Fig. 4f) [221, 222]. For instance, miR-10a interacts with the 5′UTR of mRNAs encoding ribosomal proteins and causes an increase in translation [141]. Another example is miRNA-mediated translation activation, which is associated with Ago and AU-rich elements (AREs) [221]. One of the ARE-binding proteins, HuR, is able to derepress miRNA-mediated translation inhibition (Table 1) [223]. Moreover, miRNA binding may also trigger increased ribosome loading and therefore upregulate protein synthesis [224].

### Transcript deadenylation and degradation

More recently, transcript deadenylation was demonstrated to be a widespread effect of miRNA activity (Fig. 4b) [188] and is suggested to lead to mRNA degradation (Fig. 4d, e). In specific cases, mRNA cleavage by miRISC was also reported [225, 226]. Bartel and colleagues have shown that most (at least 84 %) of the decreased protein production caused by miRNAs is an effect of reduced mRNA levels [189]. Thus, most of the research on miRNA-mediated mechanisms currently focuses on the issue of mRNA decay. Apart from Ago and GW182, several other proteins (e.g., PABP, deadenylase complexes PAN2–PAN3 and CCR4-NOT) have been demonstrated to be crucial components of the deadenylation mechanism (Table 1) (reviewed in [227]). It was proposed that deadenylation in human cells occurs in two sequential phases. First, PAN2 (the catalytic subunit of the PAN2–PAN3 complex) mediates the initial rapid deadenylation; then, two subunits of the CCR4-NOT complex, CCR4 (CNOT6/6L) and Caf1 (CNOT7/8), are most likely responsible for shortening the polyA tail [228, 229].

The mechanism of deadenylation is being carefully studied, including the structural aspects of the protein-protein interactions, to create a detailed model of deadenylation-dependent repression and better understand this process (reviewed in [230, 231]). It has been demonstrated previously that PAN3 [232] and TNRC6 possess a PAM2 motif, which enables them to interact directly with PABP (Fig. 4b) [200, 233, 234]. In addition, TNRC6 recruits PAN3 (Fig. 4b) [200, 201]. The interaction of the PAN2–PAN3 complex with PABP likely facilitates deadenylation. Conversely, no subunit of the large deadenylase complex CCR4-NOT contains a PAM2 motif, though this protein complex may cooperate with the TOB protein, which contains such a motif (Fig. 4b). In addition, CNOT1 (a scaffold and the large subunit of CCR4-NOT) interacts with TNRC6 (Fig. 4b) (reviewed in [235]).

Deadenylation may be continued by transcript degradation either via decapping by DCP1–DCP2 complex activity (Fig. 4c) and the 5′-to-3′ exonuclease XRN1 (Fig. 4d) or by 3′-to-5′ cytoplasmic exonucleases (Fig. 4e) (Table 1) (reviewed in [236]). It has been shown that decapping factors are directly recruited by PAN2–PAN3 and CCR4-NOT [200–202]. This binding might be facilitated by the high concentration of these proteins in P bodies. These cellular compartments are cytoplasmic foci where miRNAs and miRNA targets, together with proteins involved in deadenylation and decapping, are found. Moreover, translationally repressed mRNAs are stored and degraded in the P bodies ([237], reviewed in [238]). Interestingly, Izaurralde and colleagues suggested that miRISC also promotes deadenylation-independent decapping because of the indirect interaction of decapping factors with miRISC (Fig. 4i) [239].

### Translation repression may precede mRNA deadenylation and degradation

According to the previous suggestions [208, 216, 233] and recently published reports [234, 240, 241], translation regulation and mRNA decay following deadenylation are thought to be directly connected (Fig. 4j). Filipowicz and colleagues have reported that translation repression may precede deadenylation and mRNA decay [240]. Bushell and colleagues confirmed this observation and showed that translational inhibition (caused by the inhibition of initiation factor eIF4A2 binding) is the primary event required for mRNA degradation [241]. Izaurralde and colleagues conducted functional assays on *D. melanogaster* and human cells, which indicated that miRNA-mediated translational repression and degradation are mechanistically linked through the interactions of GW182 proteins with PABP and deadenylases [234]. It could be expected that this link between translation inhibition and deadenylation might function to modulate transcript turnover.

### miRNA activity in the nucleus

Following the trend of epigenetic research, increasing attention has recently been paid to short non-coding RNAs (i.a., siRNA, miRNA) that may regulate transcription (reviewed in [242, 243]). Interestingly, Meister and colleagues identified heterogeneous nuclear ribonucleoprotein particles, i.e., hnRNP-U and hnRNP-F, among proteins immunoprecipitated with Agos [220], which indicates the possible interaction of these proteins with Agos in the nucleus. Moreover, it has been shown that importin-8 (Imp8) is an essential protein factor required for the miRNA-mediated regulation of gene expression and for the localization of Ago2 in the nucleus (Table 1) [244].

Surprisingly, a recently published work described a TNRC6A paralog that possesses both a nuclear localization signal (NLS) and nuclear export signal (NES) [245]. These sequences allow TNRC6A to function as a navigator for Ago proteins into and out of the nucleus by Exportin-1 (Table 1) [245]. Also, Dicer was found in the nucleus [246], and a non-canonical NLS was recently identified within its C-terminal region [247]. However, the role of Dicer in the nucleus remains unclear.

Taken together, these results strongly suggest the importance of Ago protein localization and activity in the nucleus (Fig. 4g). Thus far, Ago complexes in the nucleus are thought to play a major role in the RNA-mediated alternative splicing process involving chromatin remodeling (Fig. 4h) [248, 249]. In agreement with these observations, high-throughput analysis of the miRNA interactome by CLASH identified targets mapped to splice junctions [131]. Moreover, the deadenylase complex CCR4-NOT, interacting with miRISC, is proposed to play an auxiliary role in transcription elongation [250]. Nevertheless, the detailed mechanism of miRNA involvement in transcription regulation is poorly understood.

Based on the results described above and the widespread effect of chromatin remodeling on the regulation of gene expression, we anticipate that future research will reveal more examples of miRNAs participating in nuclear processes.

## Implications of miRNA-mediated mechanisms for therapy

The complexity of the abundant miRNA-mRNA interactions arises from the fact that one mRNA may harbor binding sites for numerous different miRNAs [149, 251]; also, one miRNAs may be involved in regulating the expression of many transcripts. Thus, aberrant miRNA expression, either up- or downregulation, impairs cell homeostasis and is associated with a wide variety of human disorders (collected in the Human miRNA Disease Database) [252].

The precise modulation as well as reversal of such miRNA alterations is not only a challenge for fundamental research but also a promising strategy for miRNA-based therapy. Such therapy might be achieved by introducing miRNA blockers (sponges, inhibitors, anti-miRNAs) or miRNA mimics (artificial miRNAs) that may target dysregulated miRNA pathways. Recently, an efficient miRNA-based drug against hepatitis C (miRavisen, anti-miR-122) was tested in the second phase of clinical trials [253, 254].

The knowledge of miRNA-mediated mechanisms is also very useful for designing improved RNAi-based therapeutic tools. A popular trend is the use of pri-/pre-miRNA-based shRNA expression cassettes for more efficient and longer lasting gene silencing (reviewed in [255, 256]). Another example is the use of miRNA-like siRNAs for the downregulation of genes responsible for Huntington’s disease (HD) and spinocerebellar ataxia type 3 (SCA3), as recently described by Corey and colleagues as well as our group [257–260]. The principle of this approach is to directly target the mutation site, i.e., the expanded CAG repeat tract in the transcript of the mutant gene. The siRNAs that have been developed imitate miRNAs, as they form mismatched interactions with the target sequence and possess multiple neighboring binding sites on the mutant transcript. Importantly, the normal allele of the implicated transcript, as well as transcripts of other genes containing short CAG repeat tracts that are thought to provide only a single binding site for RISC, is much less efficiently silenced by miRNA-like siRNAs. Hence, the multiple RISC-binding sites allow for very efficient silencing of the mutant transcript, which might result from the cooperative action of adjacent miRISCs on the expanded CAG tract [260, 261]. It is worth highlighting that the multiple binding sites for the CAG repeat-targeting siRNAs are localized within the transcript CDS. Analyses of the silencing mechanism of these siRNAs revealed that translational inhibition is involved rather than Ago2-mediated mRNA cleavage or mRNA degradation, and a crucial role for the Ago2 and TNRC6A-C proteins was demonstrated [261].

## Conclusions and future perspectives

Although important advances have been made in miRNA research over the past several years, we still remain unable to profoundly comprehend the miRNA-based cellular processes. Numerous detailed questions remain unanswered, and among them are the following: Which factor is most important in determining the effect of miRNA activity? Is it the position of miRNA/mRNA mismatches? Is it the localization and number of miRNA-binding sites? How miRNAs within miRISCs find their binding sites on the transcript sequence is also poorly understood. Furthermore, it is not clear whether we already know all of the critical factors involved in mature miRISC formation and miRNA functioning in the cell.

As new mechanisms (e.g., alternative RISC assembly, transcriptional regulation, nuclear activity) are discovered, the question of whether they are widespread is of great importance. The results indicate that there is no single pathway of miRNA-mediated regulation common to all miRNAs in human cells. Moreover, the contradictory findings concerning the specific steps of translation that are inhibited via miRNAs remain very controversial. It seems that the variety of systems used to perform miRNA research might strongly contribute to the observed discrepancies.

Nevertheless, in agreement with recent results, the prevailing opinion concerning miRNA-mediated mechanisms is that silencing is mainly caused by transcript decay, which follows mRNA deadenylation. Thus, much effort has been made to identify the proteins involved in this process as well as interactions that take place between these proteins. The relevant information is collected in Table 1. This table presents multiple direct protein-protein interactions and suggests many indirect associations; however, it is likely that other proteins are also involved. We still do not know much about the stoichiometry of these interactions or how miRNA and transcript binding by miRISC influences protein interactions within this complex.

More generally, we consider that multiple miRNA-mediated mechanisms may operate in cells depending on, i.e., the nature of the miRNA-mRNA interactions and the type of cell or its physiological state. In a single cell, there are abundant interactions among mRNAs, miRNAs and their variants as well as various protein factors competing for interactions with miRISC. Therefore, the notion that the specific miRNA/mRNA duplex may activate not one but two or more different silencing mechanisms concomitantly (e.g., deadenylation and translation inhibition) cannot be ruled out.

Over the last few years, a growing body of evidence has formed that supports the existence of non-canonical miRNA-binding sites, in a sense of: miRNA pairings with targets, localization within the CDS and interactions with noncoding RNAs. This notion needs to be implemented into next-generation MRE prediction algorithms and taken into account in analyses of relevant deep sequencing data. Also worth highlighting is the fact that miRNA binding to non-canonical targets can modulate the levels of miRNAs and their canonical functions. Together, it is also likely that additional new functions of miRNAs await to be disclosed.

## Abbreviations

Ago: Argonaute
ARE: AU-rich element
CLIP: UV crosslinking and immunoprecipitation
CLASH: Crosslinking, ligation and sequencing of hybrids
DGCR8: DiGeorge syndrome critical region gene 8
Exp1: Exportin-1
Exp5: Exportin-5
Imp8: Importin-8
HuR: Human antigen R
miRISC: miRNA induced silencing complex
miPDC: miRNA precursor deposit complex
miRNA: MicroRNA
MRE: microRNA response element
NES: Nuclear export signal
NLS: Nuclear localization signal
ncRNA: Non-coding RNA
P-body: Processing body
PABP: PolyA-binding protein
PACT: Protein activator of PKR kinase
pri-miRNA: miRNA primary transcript
pre-miRNA: miRNA precursor
RBP: RNA-binding protein
RLC: RISC-loading complex
RNP: Ribonucleic acid protein complex
RRM: RNA recognition motif
siRNA: Small interfering RNA
TRBP: HIV-1 transactivation response RNA-binding protein
